# Retinal Functional and Structural Changes in the 5xFAD Mouse Model of Alzheimer’s Disease

**DOI:** 10.3389/fnins.2020.00862

**Published:** 2020-08-13

**Authors:** Jeremiah K. H. Lim, Qiao-Xin Li, Zheng He, Algis J. Vingrys, Holly R. Chinnery, Jamie Mullen, Bang V. Bui, Christine T. O. Nguyen

**Affiliations:** ^1^Department of Optometry and Vision Sciences, University of Melbourne, Parkville, VIC, Australia; ^2^Optometry and Vision Science, College of Nursing and Health Sciences, Flinders University, Bedford Park, SA, Australia; ^3^Florey Institute of Neuroscience and Mental Health, Parkville, VIC, Australia; ^4^AstraZeneca Neuroscience, Cambridge, MA, United States

**Keywords:** retina, Alzheimer’s disease, electroretinography, optical coherence tomography, mouse

## Abstract

Alzheimer’s disease is characterized by the aberrant deposition of protein in the brain and is the leading cause of dementia worldwide. Increasingly, there have been reports of the presence of these protein hallmarks in the retina. In this study, we assayed the retina of 5xFAD mice, a transgenic model of amyloid deposition known to exhibit dementia-like symptoms with age. Using OCT, we found that the retinal nerve fiber layer was thinner in 5xFAD at 6, 12, and 17 months of age compared with wild-type littermates, but the inner plexiform layer was thicker at 6 months old. Retinal function showed reduced ganglion cell responses to light in 5xFAD at 6, 12, and 17 months of age. This functional loss was observed in the outer retina at 17 months of age but not in younger mice. We showed using immunohistochemistry and ELISA that soluble and insoluble amyloid was present in the retina and brain at all ages. In conclusion, we report that amyloid is present in brain and retina of 5xFAD mice and that the pattern of neuronal dysfunction occurs in the inner retina at the early ages and progresses to encompass the outer retina with age. This implies that the inner retina is more sensitive to amyloid changes in early disease and that the outer retina is also affected with disease progression.

## Introduction

Alzheimer’s disease (AD) is the most common cause of dementia worldwide. The disease is characterized by abnormal processing and clearance of beta-amyloid (Aβ) and tau protein leading to the formation of Aβ plaques and neurofibrillary tangles in the central nervous system (Hardy and Higgins, 1992). These neurotoxic proteins have been shown to contribute to multiple pathological processes involving neuronal inflammation as evidenced by microgliosis and astrocytosis, followed by oxidative stress, mitochondrial dysfunction, impaired synaptic transmission, and neuronal apoptosis (Musiek and Holtzman, 2015; Selkoe and Hardy, 2016). Clinically, a range of symptoms most typically characterized by progressive memory loss and a decline in executive function (Wang et al., 2017) is observed. A report by the World Health Organization estimates that the average survival from disease onset is 4.6 years, with a median of 7.1 years (WHO, 2012). Whilst the disease is formally diagnosed through a histopathological examination post-mortem, Aβ biomarkers such as positron emission tomography and cerebrospinal fluid assays have been shown to detect the disease up to 20 years before clinical onset (Masters et al., 2015). As these tests are either invasive or difficult to access outside of research institutions, there has been a move toward developing more accessible biomarkers.

Anatomically, the sensory retina and neurons of the cortex have a common developmental origin and share many structural and functional similarities (Wallace, 2011). This opens the possibility of using the retina as a biomarker for cortical diseases such as dementia (Guo et al., 2010; Lim et al., 2016; Nguyen et al., 2017). Epidemiological surveys suggest that patients with early stage dementia show changes in the neural retina, such as thinning of retinal ganglion cell axons in the retinal nerve fiber layer (Ascaso et al., 2014; Cheung et al., 2015; Ko et al., 2016). This thinning and the associated inner retinal dysfunction (Katz et al., 1989; Trick et al., 1989; Parisi et al., 2001) has been shown to correlate with mini-mental state scores (Ko et al., 2016) in those with AD. These studies highlight the possibility that AD pathology might produce a specific pattern of retinal structural and functional deficits. This has since lead to a rapid increase in studies searching for viable ocular biomarkers of AD.

Although some studies have been able to detect Aβ in human AD retinae (La Morgia et al., 2016; Koronyo et al., 2017; Grimaldi et al., 2019), others have failed to confirm the presence of retinal plaques (Schon et al., 2012; Ho et al., 2013; Williams et al., 2017). In murine models of AD that overexpress Aβ in the brain, studies have also found that Aβ is present in the retinae of a variety of strains such as the Tg2576 (Dutescu et al., 2009; Liu et al., 2009; Alexandrov et al., 2011), APP/PS1 (Ning et al., 2008; Perez et al., 2009; Alexandrov et al., 2011; Gupta et al., 2016), 3xTg (Alexandrov et al., 2011; Grimaldi et al., 2018) TgCRND8 (Buccarello et al., 2017) TgF344 (Tsai et al., 2014), and 5xFAD (Alexandrov et al., 2011). In APP/PS1 (Ning et al., 2008; Gupta et al., 2016) and Tg2576 (Dutescu et al., 2009; Liu et al., 2009) mice, Aβ deposits were found in the inner retina near the output cells of the eye, the ganglion cells and their dendrites and axons. Quantitative comparisons in 3xTg mice indicate similar findings of early inner retinal Aβ-plaque deposition followed by later outer retinal deposits (Grimaldi et al., 2018). Whether mouse models of retinal Aβ also exhibit both *in vivo* structural and functional correlates remains incompletely investigated.

Human studies indicate a predilection of retinal ganglion cell related losses in AD patients. This is supported by deposition of Aβ in inner retinal layers (La Morgia et al., 2016; Koronyo et al., 2017) as well as losses in pattern electroretinography and retinal nerve fiber layer (RNFL) thinning with optical coherence tomography (OCT), reviewed elsewhere (Thomson et al., 2015; Lim et al., 2016; den Haan et al., 2017; Nguyen et al., 2017; Chan et al., 2019). Ganglion cell inner plexiform layer (GCIPL) thinning has also been reported (Chan et al., 2019), however, early in the disease thickening may also occur in this layer (Snyder et al., 2016). In contrast, electroretinography (ERG) assessment in mouse models of Aβ deposition at times show conflicting results. Some studies indicate for an improvement in ERG with APP/PS1 (Joly et al., 2017) and 3xTg mice (Chiquita et al., 2019) whereas others indicate for retinal dysfunction in APP/PS1 (Perez et al., 2009; Gupta et al., 2016; Georgevsky et al., 2019). It is possible in the APP/PS1 mice that this may reflect a difference in ages and ERG parameters assessed, however, some controversy still remains. OCT changes in transgenic mouse models indicate for consistent inner retinal thinning, however, this is preferential [TgCRND8 mice (Buccarello et al., 2017)] or in parallel to outer retinal changes [APP/PS1 (Georgevsky et al., 2019), 3xTg mice (Chiquita et al., 2019)]. Importantly only two studies have investigated both ERG and OCT in a murine model (Chiquita et al., 2019; Georgevsky et al., 2019). What is currently lacking is an understanding of the time course of structural changes and their corresponding function, particularly in the inner retinal layers, which is more commonly reported in humans.

The work of Criscuolo et al. (2018) indicates the 5xFAD model may be a useful one to study vision changes which recapitulate human AD as functional measures indicate that retinal ganglion cell derived pattern ERG declines faster than middle/outer retinal full field ERG responses. We extend this work in 5xFAD mice by studying whether inner retinal functional changes in these mice are mirrored by structural changes with increasing age. These mice have undergone quantitative immunohistochemical (IHC) analyses and indicate for the presence of both Aβ oligomers and Aβ plaques in the retinae and brains (Habiba et al., 2020). The current study extends this by using ELISA with qualitative IHC to examine the age-related pattern of soluble and insoluble Aβ and how this reflects on retinal structure and function in the 5xFAD mouse model.

## Materials and Methods

### General Procedures

All procedures were conducted in accordance with the National Health and Medical Research Council Australian Code of Practice for the care and use of animals for scientific purposes. Ethics approval was obtained from the Howard Florey Institute Animal Experimentation Ethics Committee (Approval number 13-068-UM). Mice used in this study B6.CgTg (APPSwFlLon,PSEN1*M146L*L286V) 6799Vas/Mmjax, carry three human APP mutations and two PSEN mutations, hence termed “5xFAD.” In this model, amyloid expression is driven by a neuron-specific Thy1 promoter, allowing for the accumulation of high levels of Aβ42 in the cortex, rapidly recapitulating the features of AD which include amyloid plaque formation in the cortex and behavioral changes such as memory impairment (Oakley et al., 2006). The strain used in this study (MMRRC Stock 34848, Jackson Laboratories, Bar Harbor, ME, United States) are segregating hemizygous/wildtype for the disease and are bred on a C57BL/6J background and backcrossed such that they do not carry the retinal degeneration allele Pde6b^rd1^. Due to the congenic background of these mice, non-transgenic littermates (WT) were used as a control (Oakley et al., 2006).

Mice were examined at three ages, namely 6, 12, and 17 months of age. A total of 32 5xFAD and 38 WT mice were used. Each age group underwent non-invasive assessment of retinal structure and function on separate occasions. Before each procedure, animals were weighed and anesthetized with an intraperitoneal injection of ketamine (80 mg/kg) and xylazine (10 mg/kg). The mixture was diluted in sterile injectable saline (1:10) to aid with hydration and ease of administration (10 μl/g).

Topical anesthesia and pupil mydriasis were achieved with drops of proxymetacaine 0.5% and tropicamide 0.5% (Alcaine^TM^ and Mydriacyl^TM^, respectively, Alcon Laboratories, Frenchs Forest, NSW, Australia). Corneal hydration was maintained with either lubricating eye drops or eye gel (Systane^®^ or Genteal^®^, Novartis Pharmaceuticals Australia). At the end of the final experiment, anesthetized animals were culled by cervical dislocation. Eye and brain tissues were collected *postmortem* for assessment.

### Electroretinography

Mice were dark adapted overnight prior to ERG recording (5xFAD, *n* = 11–15/age; WT, *n* = 8–12/age). As previously described (Nguyen et al., 2016; Zhao et al., 2017), experiments were conducted in a lightproof room with the aid of a dim red light in order to preserve dark adaptation. Upon induction of anesthesia and mydriasis, animals were lightly secured to a heated platform with straps to minimize movement and breathing artifacts. A custom made chlorided silver active electrode (A&E Metal Merchants, NSW, Australia) was placed upon the central cornea, with the inactive placed around the sclera and the reference electrode (Grass Telefactor, RI, United States) inserted subcutaneously into the tail, before positioning the Ganzfeld bowl at eye level. ERGs were elicited using a range of increasing luminous energies. This range of stimuli allows for the isolation of ganglion cell (scotopic threshold response, STR), inner retinal inhibitory circuits (oscillatory potentials, OPs), bipolar cell (P2), and photoreceptor (P3) response amplitudes and timings. ERG parameters returned from the analysis include the P3 (RmP3), P2 (Vmax), OP, and pSTR amplitudes, the P3 and P2 sensitivities, in addition to the OP and pSTR implicit times.

Analysis of the ERG has been described in detail previously (Nguyen et al., 2016; Zhao et al., 2017). In brief, the first electronegative component of the ERG waveform was modeled using a delayed gaussian function to expose the P3. This is subtracted from the waveform to isolate the P2-OP complex. The OPs are separated from the P2 by transforming the data into the frequency domain via discrete fourier transform, followed by the filtering using a digital band pass filter (50–180 Hz, −3 dB). The STR parameters are averaged over three luminous energies, −4.90, −5.01, and −5.31 log cd⋅s/m^2^ in order to assess retinal ganglion cell function.

### Optical Coherence Tomography

Retinal structure was measured (5xFAD, *n* = 8–14/age; WT, *n* = 8–16/age) using spectral domain-OCT (Envisu-R2200, Leica Microsystems, Buffalo Grove, IL, United States). Retinal volumes (1.4 × 1.4 × 1.57 mm) centered at the optic nerve head were acquired using 200 evenly distributed horizontal B-scans, each made up of 1000 A-scans. This yielded a volume with a lateral resolution of 7 μm superiorly to inferiorly, with an axial depth resolution of 2.8 μm. For analysis, images were extracted as a TIFF stack and quantified using FIJI software (National Institutes of Health, Bethesda, MD, United States). Retinal layers in the central b-scan were manually segmented in a masked fashion into the retinal nerve fiber layer (RNFL), ganglion cell complex (GCC), and total retinal thickness (TRT) components. The difference between GCC and RNFL was taken to be the inner plexiform layer (IPL), whilst the difference between TRT and GCC was taken to be the outer retinal layer (ORT).

### Immunohistochemistry

Mice were perfused with heparinized phosphate buffered saline. Whole eye and brain tissues were dissected and fixed in 10% neutral buffered formalin overnight and embedded in paraffin for sectioning. Sagittal sections 7 μm in thickness were cut and treated with 80% formic acid and 3% hydrogen peroxide prior to incubation in blocking buffer (50 mM Tris-HCl, 175 mM NaCl, pH 7.4, with 20% blocking serum corresponding to species for secondary Aβ). In the brain a total of 135 sections were obtained while 1272 retinal sections were obtained from 6 5xFAD mice and 3 WT. Monoclonal antibodies 1E8 and WO2 were obtained in-house and have been previously validated (Ida et al., 1996; Allsop et al., 1997; Li et al., 1998; Tammer et al., 2002; George et al., 2006). They were used at dilutions of 1:500 and 1:1000, respectively. Sections were washed and incubated with biotinylated secondary antibody and streptavidin/horseradish peroxidase reagent (Dako LSAB^®^ + HRP kit, Agilent Technologies Australia, Mulgrave VIC, Australia), followed by chromogen for color development (Dako DAB + chromogen kit, Agilent Technologies Australia, Mulgrave VIC, Australia). Slides were counterstained in Harris’s Haematoxylin (Australian Biostain, Traralgon, VIC, Australia), before mounting in distyrene-plasticizer-xylene media (DPX new, Merck Millipore, Bayswater, VIC, Australia). Before each run, a sample whereby the primary antibody was removed served as a negative control. IHC staining of frontal cortex and hippocampus were quantified as described by Wilcock et al. (2006) In brief, amyloid deposits were first thresholded in a masked fashion according to a fixed hue-saturation-intensity used across all images of the same magnification. In order to incorporate both size and number of Aβ plaques in a given image, the stain area is quantified as a percentage of the total area of the given image. Three sections at each location from each mouse (6 months 5xFAD *n* = 3, WT *n* = 1; 12 months 5xFAD *n* = 3, WT *n* = 1, 17 months 5xFAD *n* = 3, WT *n* = 1) was assessed and averaged into a single parameter for each individual animal.

### Protein Assay

Eye and brain tissues were snap frozen in liquid nitrogen immediately after collection. Eyes were dissected with the aid of a microscope in order to retrieve the retinae. In short, an eye cup was created by cutting circumferentially around the limbus. The lens and vitreous were removed with tweezers and four relaxing incisions were made to the globe to yield a flat mount. The retinal tissue was then isolated by gently leveraging it from the sclera with a Tooke knife (AS4-020, Aurora Surgical LLC, St Petersburg, FL, United States). Due to the small volume of retinal tissue, which is estimated to be ∼10 mg when wet (Okawa et al., 2008), and in accordance with pilot studies 6–8 retinae of the same age group and genotype were pooled into one sample for protein assays. Brains were assayed individually.

Each sample was homogenized in either 1000 μl (brain) or 200 μl (retina) of saline buffer containing a protease/phosphotase inhibitor (Halt^TM^ Protease and Phosphatase Inhibitor Cocktail 100X, ThermoFischer, Scoresby, VIC, Australia) using a sonicator (Branson digital sonifier, Model S450, Danbury, CT, United States) with 30 second bursts on ice until tissues were visibly homogenized. As previously described (McLean et al., 1999; Adlard et al., 2014; Roberts et al., 2017), the homogenate was then separated using an ultracentrifuge (OptimaTM Max-E, Beckman Coulter Australia, Lane Cove, NSW, Australia) at 100,000 × *g* on a 1-h spin cycle at 4°C. The resulting supernatant was analyzed for “soluble” protein. The pellet was re-suspended in protease inhibitor buffered saline and homogenized again to create the “insoluble” protein sample. Samples were then aliquoted for protein analysis described below.

#### Total Protein Quantification Using Bicinchoninic Acid Assay

Total protein levels of the soluble and insoluble samples were determined with bicinchoninic acid (BCA) assay (Pierce Biotechnology, Rockford, IL, United States). Aliquots (5 μl) of soluble eye and brain samples were diluted in dH_2_O at a ratio of 1:10 and 1:20. Insoluble eye and brain aliquots (200, 1000 μl, respectively) were diluted at ratios of 1:20 and 1:40. Once diluted, 10 μl samples were added into well plates containing 200 μl of BCA working buffer and incubated at 37°C for 30 min. Once developed, the samples, along with known standard albumin concentrations were evaluated on a plate reader (Wallac 1420 Victor^2^ microplate reader, Perkin Elmer, Waltham, MA, United States) using a 560 nm source. Standard concentrations values were used to derive a standard curve in order to determine the final total protein concentration of the samples. Samples were run in duplicate to reduce variability.

#### Amyloid Protein Quantification Using ELISA

Enzyme-linked immunosorbent assay was performed for quantification of total Aβ using W02 primary antibody-coated well plates. After washing, the samples were blocked with 0.5% casein/PBS and washed, prior to the addition of 10 μl of detection antibody, 1E8-Biotin (2 ng/μl) into each well. Aβ40 standards (50 μl) with known concentration were made up using serial dilution in order to establish the standard curve. Soluble (50 μl) samples were directly loaded into each well. Insoluble 5 μl aliquots were first diluted in 15 μl formic acid (1:4) and left to solubilize for 1 h, followed the addition of 1 M Tris (1:20) and PBS/casein (1:5); before being loaded into each well at 50 μl volumes. Samples were then incubated overnight at 4°C. After washing, Streptavidin-Europium (Delfia^®^ 1244-360, Perkin Elmer, Boston, MA, United States) in 1:1000 PBST/casein was added and the solution was incubated for 1 h at room temperature. After washing, enhancement solution (Delfia^®^ 1244-105 Perkin Elmer, Boston, MA, United States) was added to each well before being loaded into the plate reader. Plates were read using excitation at 340 nm and emission at 650 nm in order to determine Aβ concentration. 5xFAD and wild type tissue were assayed and the reliable detection limit was determined by loading tissue from the soluble and insoluble fraction of the controls. The transgenic retinal tissue was above this limit and illustrated in Figure 3. Results are expressed as Aβ (pg) per mg of total protein as determined using BCA. To facilitate comparison between retina and brain samples Aβ levels are normalized to protein concentration (in mg).

### Data Analysis and Statistics

Statistical comparisons were carried out using two-way ANOVA to establish differences between treatment and time effects. Two-way ANOVA *post hoc* tests with Bonferroni correction for multiple comparisons were used to compare between groups. Normality was established using a Kolmogorov-Smirnov test. An alpha of 0.05 was considered to be statistically significant. Statistical analyses were carried out in Prism 6 (GraphPad, La Jolla, CA, United States).

## Results

### Immunohistochemistry

#### Amyloid Deposits in the Brain Increases With Age

**FIGURE 1:**
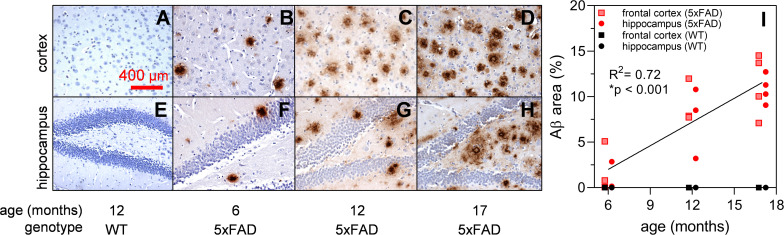
Expression of Aβ in 5xFAD mouse brain at 6, 12, and 17 months of age. Aβ labeling using monoclonal mouse antibody 1E8 (brown) and counterstained with haematoxylin (blue). Representative cortical and hippocampal parasagittal serial sections of 12-month-old wild type **(A,E)**; and 5xFAD mice at ages 6 **(B,F)**, 12 **(C,G)**, and 17 **(D,H)** months of age. There is a significant increase in Aβ area stained with advancing age in 5xFAD mice **(I)** *Scale bar, 400 μm.*

Brain sections of 5xFAD and wildtype (WT) mice at 6, 12, and 17 months of age underwent immunohistochemical (IHC) staining using Aβ specific 1E8 antibodies (Dutescu et al., 2009; Figure 1). Background structures were outlined using haematoxylin stains. Figures 1A–H show representative images where Aβ immunoreactivity increases with age (brown staining). This is further quantified for the percent area of Aβ staining (Figure 1I) and illustrates an increase in the 5xFAD sections (*R*^2^ = 0.72, *p* < 0.001) but not age-matched controls.

#### Discrete Amyloid Deposits Were Rare but Detectable in the Retinae of 5xFAD

**FIGURE 2:**
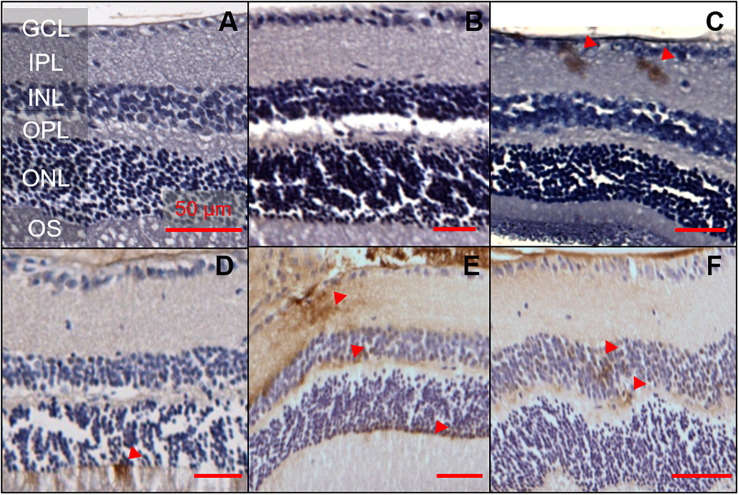
Aβ was detectable in 5xFAD mice retinae at various ages. 1E8 labeled serial sections counterstained with haematoxylin showed Aβ immunoreactivity in the inner and outer retina of 5xFAD mice. 1E8 staining absent in **(A)** a 6-month-old WT mouse retina and **(B)** a negative control in a 6-month-old 5xFAD retina. 1E8 staining present in **(C)** a 6-month-old 5xFAD retina **(D)** a 12-month-old 5xFAD retina and **(E,F)** a 17-month-old 5xFAD retina. Positive Aβ staining was seen in the ganglion cell layer, inner plexiform layer, the inner and outer nuclear layers as marked by red arrows. Images feature tangential cuts to the retina which preclude meaningful thickness comparison. Scale bars, 50 μm; GCL, ganglion cell layer; IPL, inner plexiform layer; INL, inner nuclear layer; OPL, outer plexiform layer; ONL, outer nuclear layer; OS, outer segments. Disclosure: separate histological sections from a subset of these animals have been displayed in another manuscript (Hadoux et al., 2019).

The same 1E8 antibody and IHC staining protocol was used in the retina. A total of nine animals, aged 6, 12, and 17 months were examined (two 5xFAD and one WT animal per age group, Figure 2). Of the six 5xFAD animals, only four animals aged 6 (*n* = 1), 12 (*n* = 1), and 17 months (*n* = 2) showed Aβ reactivity in the retina. None of the WT mice examined (165 sections across 3 WT retinae) were positive for Aβ.

Detection of Aβ staining in retinal serial sections (7 μm) of 5xFAD mice proved elusive, with only 16 out of the 1,107 sections (1.4%) examined showing positive Aβ staining. There appears to be a preference for the staining (50%, 8 of 16 sections) to occur in the ganglion cell layer (GCL) and inner plexiform layer (IPL, Figure 2). The remaining 50% of the staining was found in the inner nuclear layer (INL, *n* = 1), outer plexiform layer (OPL, *n* = 1) and outer nuclear layer (ONL, *n* = 6). In 6-month-old 5xFAD, retinal staining was limited to the inner retinal layer, with no immunoreactivity in the outer retina.

### Amyloid Protein Assay

#### Amyloid Quantification Using ELISA Shows Increased Protein Expression With Age

**FIGURE 3:**
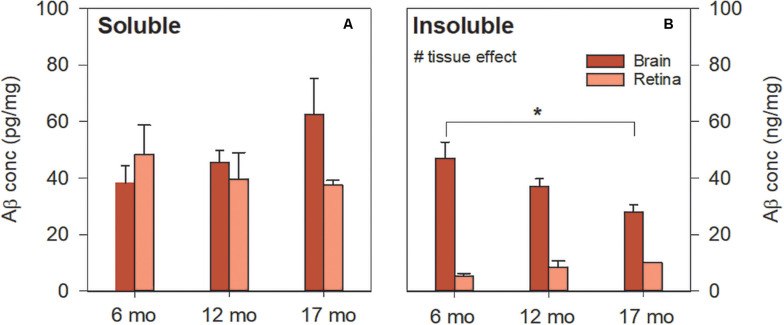
Aβ levels in 5xFAD brain and retina using ELISA. **(A)** Soluble Aβ in the brain increased with age but remained unchanged in the retina with age. Soluble Aβ levels in the retina were comparable to that of the brain. **(B)** Insoluble Aβ levels in the retina were lower than that of the brain. Aβ was not detected in WT mice (not shown). Bars, Mean ± SEM; ^#^*p* < 0.05 for tissue effect on two-way ANOVA analyses. ^∗^*p* < 0.05 for Bonferroni *post hoc* tests.

Soluble Aβ in the brain and retina showed similar levels [Figure 3A, two-way ANOVA *F*_(1, 41)_ = 0.55, *p* = 0.46]. In contrast, Figure 3B illustrates the difference in insoluble Aβ between the retina and the brain at each age. Here, we found that insoluble retinal Aβ was significantly lower than brain levels [two-way ANOVA, tissue effect, *F*_(1, 41)_ = 22.47, *p* < 0.01] which may account for the relative sparsity of amyloid deposits in retinal sections examined with IHC (Figure 2). *Post hoc* analysis (Bonferroni test, 6 vs. 17 months, *p* < 0.05) indicates for a reduction in insoluble Aβ in the brain with advancing age. This is counter to our *a priori* expectation as IHC showed an age-related increase in Aβ density in the cortex of these mice (Figure 1).

### *In vivo* Retinal Structure in 5xFAD Mice

#### Optical Coherence Tomography Reveals Selective Inner Retinal Thinning in 5xFAD

**FIGURE 4:**
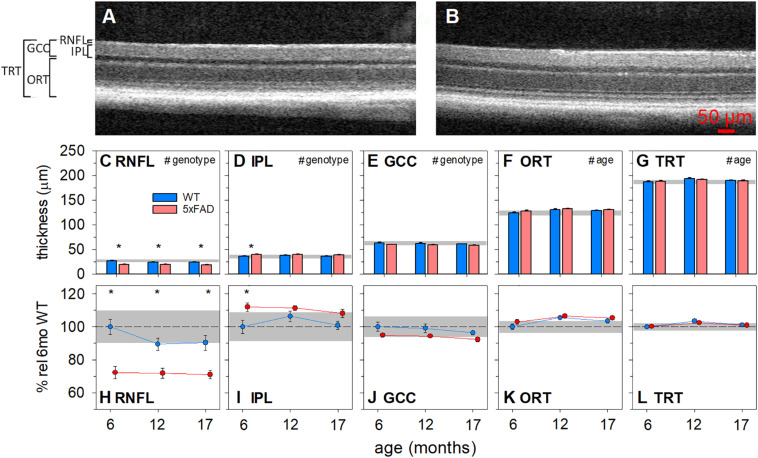
Age-related structural changes in 5xFAD examined using optical coherence tomography. Mouse retinas were assayed for structural changes at 6 (5xFAD *n* = 8, WT *n* = 13), 12 (5xFAD *n* = 12, WT *n* = 13) and 17 months (5xFAD *n* = 14, WT *n* = 16) of age and genotype differences were compared. **(A,B)** Representative WT and 5xFAD OCT images, respectively. **(C–G)** Raw retinal thickness values. **(H–L)** Retinal thickness values expressed as a percentage of 6-month-old WT. RNFL and GCC thickness were significantly reduced and IPL was significantly increased in 5xFAD mice. No significant changes were found in ORT or TRT. Scale bar, 50μm; RNFL, retinal nerve fiber layer; IPL, inner plexiform layer; GCC, ganglion cell complex; ORT, outer retinal thickness; TRT, total retinal thickness; Error bars, SEM; Gray shaded area, 95% CI for 6-month-old WT; ^#^*p* < 0.05 for treatment effect on two-way ANOVA analyses; ^∗^*p* < 0.05 for Bonferroni *post hoc* tests.

Optical coherence tomography (OCT) allows for the *in vivo* assessment of specific retinal layer thicknesses, avoiding potential confounds associated with tissue preparation and fixation (Ferguson et al., 2014). Both 5xFAD and WT controls were assayed at 6 (5xFAD *n* = 8, WT *n* = 13), 12 (5xFAD *n* = 12, WT *n* = 13) and 17 months of age (5xFAD *n* = 14, WT *n* = 16). Figure 4 shows representative OCT images, raw layer thickness values, and layer thickness expressed relative to 6-month-old WT littermates.

The RNFL layer was significantly thinner in 5xFAD mice compared with WT animals [Figures 4C,H, two-way ANOVA, genotype effect, *F*_(2, 70)_ = 38.90, *p* < 0.01]. There were no significant interactions or age effects. Bonferroni *post hoc* analysis showed that the RNFL thinning in 5xFAD mice was significant at all ages assessed (6 months, *p* < 0.01; 12 months, *p* < 0.05; 17 months, *p* < 0.01). Similarly, ganglion cell complex (GCC) thickness, which includes RNFL, ganglion cell bodies and their synaptic layer (the inner plexiform layer, IPL), was reduced in 5xFAD compared with WT [Figures 4E,J, two-way ANOVA, genotype effect, *F*_(1, 70)_ = 7.64, *p* = 0.01]. No significant interaction or age effect was found.

When compared between genotypes, the IPL in 5xFAD mice was significantly thicker than WT mice [Figures 4D,I, two-way ANOVA, genotype effect, *F*_(1, 70)_ = 11.43, *p* < 0.01]. This was especially so in the 6-month-old group (Bonferroni, 6 months, *p* < 0.01). Thus, the amount of RNFL thinning outweighed that of IPL thickening, which manifest as an overall thinning of the GCC.

Outer retinal thickness (ORT) was defined by the difference between the total thickness of the retina minus the inner retinal layers (GCC). This area represents a combination of layers where the cell bodies of photoreceptors, horizontal cells, bipolar cells and amacrine cells and their synaptic layers exist. There was a slight but significant age-related increase in ORT [two-way ANOVA, age effect, *F*_(1, 70)_ = 7.64, *p* < 0.01]. However, there was no significant interaction or genotype differences.

Total retinal thickness (TRT) provides a measure of age and phenotype effects on the retinal as a whole and allows for the comparison of the relative magnitude of change between the inner and outer retinal layer. In this instance, both genotypes showed a small increase in TRT with age [two-way ANOVA, age effect, *F*_(1, 70)_ = 6.75, *p* < 0.01], with no significant interaction or genotype differences.

### Retinal Function in 5xFAD Mice

**FIGURE 5:**
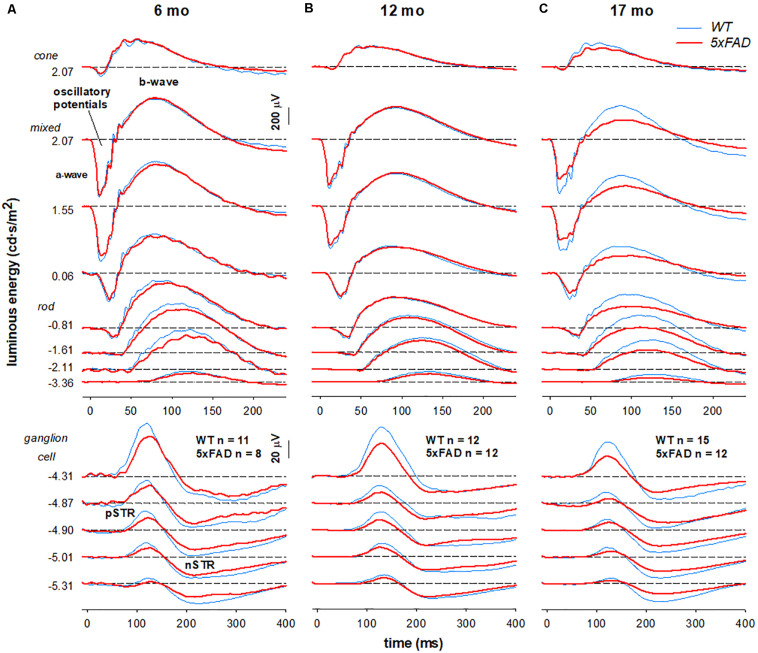
Age-related changes in retinal function in 5xFAD mice. Grouped averaged waveforms are shown for **(A)** 6-month-old **(B)** 12-month-old **(C)** 17-month-old mice. Lower panels show the ganglion cell dominated STR response (−5.01 to −4.87 log cd⋅s/m^2^). Rod and mixed rod-cone response are elicited with increasing luminous energies. The uppermost panel reflects the cone response isolated using a twin-flash paradigm. STR, scotopic threshold response – ganglion cell dominant; pSTR, positive STR; nSTR, negative STR; a-wave – photoreceptoral response; b-wave – bipolar cell response; oscillatory potentials – amacrine cell dominant.

Group averaged electroretinogram (ERG) waveforms (age 6, 12, 17 months: 5xFAD *n* = 8, 12, 12, respectively; WT *n* = 11, 12, 15, respectively) are shown in Figure 5. The ganglion cell dominated scotopic threshold response (STR) elicited with the dimmest flashes is shown in the lowest traces. Rod dominant responses are elicited for luminous energies up to ~−0.8 log cd⋅s/m^2^. For brighter stimuli, the ERG is a mixed signal containing contributions from both rod and cone pathways. In WT mice, waveforms were attenuated with advancing age. This attenuation appeared to be consistent for all ERG components.

When comparing 5xFAD and WT, attenuation of the STR response (Figure 5A, bottom panels −5.01—4.87 log cd⋅s/m^2^) was seen at 6 months of age in 5xFAD mice. At this age, ERG responses arising from the outer retina appeared relatively normal. This pattern of functional deficits was also observed in 12-month-old 5xFAD mice (Figure 5B). However, by 17 months of age reduced inner retinal function in 5xFAD mice is also accompanied by outer retinal dysfunction as evident in deficits in responses elicited with medium to higher stimulus energies (Figure 5C, −0.81–2.07 log cd⋅s/m^2^).

**FIGURE 6:**
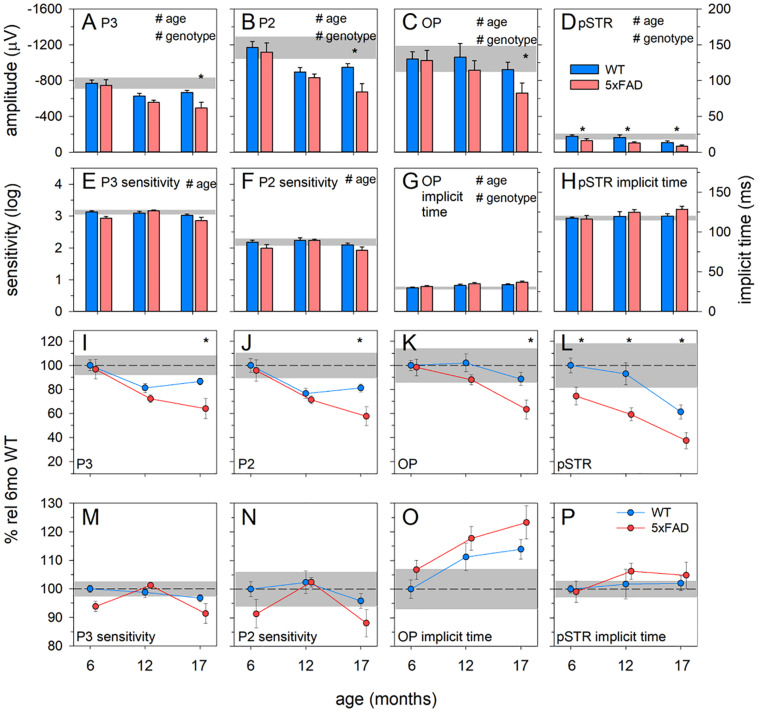
Age-related change in 5xFAD and WT ERG parameters. **(A–D)** Raw P3, P2, OP, and pSTR, respectively. **(E,F)** P3 and P2 sensitivity (units, log cd-1.s-1.m^2^), respectively. **(G,H)** OP and pSTR implicit time (ms), respectively. **(I–P)** All ERG parameters normalized to 6-month-old WT to facilitate easier comparison between parameters. The earliest functional change to occur in 5xFAD occurred in the pSTR. An overall trend toward ERG decline with age occurred in all parameters. At 17 months of age, 5xFAD mice showed a significant decline in all ERG parameters. P3, photoreceptoral response; P2, bipolar cell response; OP, oscillatory potentials; pSTR, positive scotopic threshold response; all data shown, mean ± SEM; ^#^*p* < 0.05 for treatment effect on two-way ANOVA analyses; ^∗^*p* < 0.05 for Bonferroni *post hoc* tests.

These observations are confirmed in analysis of the ERG components (Figure 6). Photoreceptoral amplitudes declined with age and were smaller in 5xFAD mice [Figures 6A,I, P3, two-way ANOVA, age effect, *F*_(2, 64)_ = 10.82, *p* < 0.01; genotype effect, *F*_(2, 64)_ = 7.26, *p* = 0.01]. Bonferroni *post hoc* analysis revealed a difference between 5xFAD and WT at 17 months of age (*p* < 0.05), but not at 6 or 12 months of age (6 months, *p* > 0.99; 12 months, *p* = 0.65).

Photoreceptoral sensitivity showed an age-related difference [two-way ANOVA, age effect, *F*_(2, 64)_ = 5.12, *p* < 0.01] but no genotype change [two-way ANOVA, genotype effect, *F*_(2, 64)_ = 3.59, *p* = 0.06]. None of the photoreceptoral parameters showed interaction effects.

Bipolar cell responses were significantly attenuated with age, with 5xFAD mice showing significant dysfunction [Figures 6B,J, P2, two-way ANOVA, age effect, *F*_(2, 64)_ = 14.09, *p* < 0.01; genotype effect, *F*_(2, 64)_ = 6.08, *p* < 0.05]. *Post hoc* analysis highlighted a difference between 5xFAD and WT at 17 months, with no difference at 6 or 12 months of age (Bonferroni, 6 months, *p* > 0.99; 12 months, *p* > 0.99; 17 months, *p* < 0.05). Bipolar cell sensitivity showed a significant change with age [Figures 6F,N,K, two-way ANOVA, age effect, *F*_(2, 64)_ = 4.98, *p* < 0.01] but no difference between genotypes [K, two-way ANOVA, genotype effect, *F*_(2, 64)_ = 4.98, *p* = 0.06]. There were no interaction effects in bipolar cell parameters.

Inner retinal inhibitory circuits involving amacrine cell function, as indicated by the amplitude of the OPs, was attenuated with age and in 5xFAD mice [Figures 6C,K, OP, two-way ANOVA, age effect, *F*_(2, 64)_ = 8.21, *p* < 0.01; genotype effect, *F*_(2, 64)_ = 7.06, *p* < 0.01]. *Post hoc* analysis showed no difference at ages 6 and 12 months but significantly smaller OPs in 5xFAD mice at 17 months of age (Bonferroni, 6 months, *p* > 0.99; 12 months, *p* = 0.33; 17 months, *p* < 0.01). OP implicit time was delayed with age [Figures 6G,O, two-way ANOVA, age effect, *F*_(2, 64)_ = 5.93, *p* < 0.01; genotype effect, *F*_(2, 64)_ = 4.38, *p* = 0.04], and was generally slower in 5xFAD mice. Similarly, ganglion cell function declined with age and was smaller in 5xFAD compared with WT mice [Figures 6D,L, pSTR, two-way ANOVA, *F*_(2, 64)_ = 17.90, *p* < 0.01 and *F*_(2, 64)_ = 26.51, *p* < 0.01, respectively]. In contrast to the other ERG parameters, the pSTR in 5xFAD mice was significantly reduced at 6 months of age. This difference persisted across all ages (Bonferroni, 6 months, *p* < 0.05; 12 months, *p* < 0.01; 17 months, *p* = 0.02). No difference in the implicit time of the pSTR was noted (Figure 6H).

In order to better visualize the relative change between inner and outer ERG parameters, data were normalized to the 6-month-old WT group and expressed as a percentage (Figures 6I–P). In WT mice, age-related decline in outer retinal responses (P3 and P2) was seen at 12 months. However, significant inner retinal decline (OPs and pSTR) was only evident at 17 months of age. In 5xFAD mice, ganglion cell dysfunction was evident at 6 months of age. Between 6 and 12 months of age outer retinal function in 5xFAD mice appeared to decline at a similar rate to WT mice. Between 12 and 17 months of age, outer retinal function (P3 and P2) along with the OPs in 5xFAD mice appeared to decline faster compared with WT mice.

## Discussion

Our study shows ERG and OCT changes that indicate for a preferential effect on the inner retina in 5xFAD mice. More specifically, we show that 6-month-old mice exhibited preferential inner RNFL thinning (Figures 4C,H) in the absence of outer retinal changes (Figures 4F,G,K,L) using OCT. This finding was, similarly, reported in 3-month-old TgCRND AD mice (Buccarello et al., 2017). To our knowledge, only two other studies have performed a time course of OCT assessment in AD mouse models, though direct comparison is limited due to the different segmentation methods used. Nevertheless, in 3xTg AD mice, Chiquita et al. (2019) reported inner retinal (GCL+IPL) thinning at the earliest time-point (4 months) before significant changes to middle and outer retinal layers (INL+OPL, ONL, and IS+OS layers) at 8, 12, and 16 months old). In APP/PS1 mice, Georgevsky et al. (2019) also examined a time-course (3, 6, 9, and 12 months old) of OCT changes and reported both inner retinal (ILM to INL) and outer retinal changes (OPL to RPE), with a trend toward greater sensitivity occurring in the inner retina. As such the early preferential inner retinal thinning is consistent across studies.

Similarly, clinical human AD studies commonly report RNFL thinning across all stages of the disease (Thomson et al., 2015; den Haan et al., 2017; Chan et al., 2019). The IPL, however, has received less attention. Snyder et al. (2016) found that in those who were at risk of AD (e.g., PET biomarker positive), there was a selective trend toward greater IPL volumes which significantly correlated with surface area of retinal inclusion bodies (suggested to contain Aβ). Similarly, Ascaso et al. (2014) found a thickening of total macula thickness with concurrent RNFL thinning in MCI patients, whereas thinning of both RNFL and macular thickness was observed in those with AD. Consistent with this, we report initial IPL thickening at 6 months (Figures 4D,I) but not at older ages. Whether this is due to amyloid deposition or gliosis resulting from inflammation (Ning et al., 2008; Perez et al., 2009) in 5xFAD mice requires further evaluation.

Functionally, 5xFAD mice show attenuation of the ganglion cell dominated pSTR, with no changes to bipolar, amacrine and photoreceptor mediated responses at 6 months (Figures 5, 6), consistent with the observed structural change in the inner retina and inner retinal amyloid deposits. In comparison with the literature, our findings were most consistent with other studies employing retinal ganglion cell measures in APP/PS1 (Gupta et al., 2016) and 5xFAD (Criscuolo et al., 2018). These ERG deficits corroborate previous reports of ganglion cell dysfunction in clinical cohorts with AD (Katz et al., 1989; Trick et al., 1989; Parisi et al., 2001; Krasodomska et al., 2010; Sartucci et al., 2010). With advancing age, we show more generalized dysfunction in 5xFAD mice, with significant reductions in P3 (photoreceptoral), P2 (bipolar cell), and OP amplitude (amacrine cell) observed by 17 months of age (Figure 6). This is in agreement with Perez et al. (2009) who found reductions in a-wave (photoreceptor) and b-wave (bipolar cell) amplitudes in 12–16 month old APP/PS1 mice. Although this did not manifest as an overall outer retinal thinning on OCT, dysfunction in cells are known to precede structural changes (Fortune et al., 2012; Fry et al., 2018; Georgevsky et al., 2019). Why other studies in 3xTg mice (Chiquita et al., 2019) and APP/PS1 mice (Joly et al., 2017) show larger outer (a-wave) and middle retinal (b-wave) responses in the rod and cone system could be explained by the difference in their functional assessment or the animal model used. This area requires further investigation. Taken together, our ERG and OCT findings suggest that in 5xFAD mice there is selective inner retina deficits at younger ages (6 months) which progress to more widespread functional deficits at older ages (17 months).

A recent study by our group has quantified immunohistochemical staining of Aβ in retina and brains in 5xFAD mice (Habiba et al., 2020). Some of the animals used in the current study also underwent tissue assessment in Habiba et al. (2020). Habiba et al. (2020) found that Aβ plaque deposition increased with advancing age in the retina, cortex and hippocampus of 5xFAD mice (6, 12 and > 14 months old). In contrast, Aβ oligomers were highest at 6 months and decreased with advancing age (12 and > 14 months old) in 5xFAD tissue. The inverse correlation between Aβ-oligomers and Aβ-plaques was consistent with an age-related conversion between the two. Analyses of age-matched wild-type controls showed higher levels in 5xFAD tissue. These patterns are largely in agreement with the soluble and insoluble ELISA conducted in the current study, however, due to the necessity to pool tissue, ELISA was less sensitive to pick up aging effects.

Alexandrov et al. (2011) compared Tg2576, 3xTg-AD, 5xFAD, and PS/APP mouse models using ELISA and found retinal Aβ in all models and highest in 5xFAD, most closely approximating that found in human retina. Extending Alexandrov et al. (2011) findings to multiple ages we found that retinal levels of the more toxic soluble form of Aβ, particularly at 6 and 12 months of age, were comparable to that of the brain (Figure 3A). In contrast, Habiba et al. (2020) found higher levels of β oligomers in the cortical and hippocampal tissue than retinal tissue which may reflect the higher specificity to Aβ oligomers of the A11 antibody than the soluble fraction of ELISA, which in addition to Aβ-oligomers also includes Aβ-monomers and dimers. Figure 3B illustrates that insoluble Aβ levels were much lower in the retina (~5–9 times lower) compared with the brain. This may explain the relative ease with which amyloid plaques were detectable using IHC staining in brain sections (Figure 1) compared with their rarity in retina (Figure 2), a finding supported by Habiba et al. (2020) and other studies using 5xFAD mice and other animal models of AD (Dutescu et al., 2009; Alexandrov et al., 2011).

Quantification of IHC in the brain shows increasing deposition of Aβ plaques with advancing age (Figure 1I) in accordance with Habiba et al. (2020) which conducted IHC using additional stains (Congo red, Thioflavin-T, 4G8). This is contrary to the findings of ELISA. One possibility was incomplete solubilization of the amyloid aggregates in the samples using formic acid treatment, which was performed over 1 hour. This could lead to epitope masking and steric hindrance, resulting in less antibody capture during ELISA (Janssen et al., 2015). This is likely to be more pronounced with older 5xFAD tissue given the likelihood of increasing plaque density (Figure 1) and hardness of the plaques (Janssen et al., 2015). This may have been less of an issue with retinal tissue, which has less Aβ deposition.

In terms of anatomical localization, the IHC staining in the current study (1E8) and that of Habiba et al. (2020) (Congo red, Thioflavin-T 4G8 A11) suggests some inner retinal preference that may extend to the outer retina with advancing age. Although the distribution of Aβ in the retina was only a qualitative observation, this is in line with the literature. Grimaldi et al. (2018) found that Aβ plaque (anti-Aβ D54D2) volume was significantly elevated in 3xTg mice particularly evident in the inner retina, increasing exponentially with age and spreading to the outer retina. Similarly, Ning et al. (2008) found an age-dependent deposition of Aβ in the RNFL layer of APP/PS1 mice. In Tg2576 mice, two studies found positive staining for Aβ using 1E8 (Dutescu et al., 2009) and 6E10, 12F4, 5C3 (Liu et al., 2009) antibodies in the inner retinal layers (Dutescu et al., 2009; Liu et al., 2009) and some in the outer retinal layers (Liu et al., 2009). As such in combination with our ERG and OCT changes, collectively this lends some credence to the idea that retinal amyloid pathology preferentially affects the inner retina, in particular the retinal ganglion cells (Blanks et al., 1989; Blanks et al., 1996).

This study has a number of limitations that are noteworthy. Although serial sections were used to detect retinal Aβ, with an interest in the retinal layer where such deposits may reside, this may have led to an underestimation of the frequency of Aβ staining. Due to the sparseness of retinal Aβ staining, perhaps the use of retinal wholemounts and confocal imaging (Park et al., 2014) would allow us to detect more Aβ accumulations in the inner retinal layer. Whilst we have shown inner retinal function loss corresponding to RNFL thinning, the individual retinal layers were not segmented or stained to show increased immunological reactivity. Studies staining for glial activation and apoptosis would be useful in the future, but is beyond the scope of the current study.

Finally, the current study found deficits in retinal function and structure even at the earliest time-point assessed (6 months of age) which corresponds to a relatively early stage of pathogenesis in this animal model. Further studies are required to examine even earlier time-points, before cortical and behavioral changes manifest which would be indicative for the retina as an early pre-clinical marker. Furthermore, this would facilitate elucidation of whether the changes seen in 5xFAD mice have developmental and/or progressive components.

Assessment of inner retinal changes with OCT and ERG as biomarkers for Alzheimer’s disease are favorable in terms of their non-invasive and inexpensive nature. OCT in particular is becoming increasingly widespread in optometric and ophthalmic clinics. Utility of these retinal assays in the field of pre-clinical and clinical assessment for developing new treatments for Alzheimer’s remains to be seen, with further studies investigating sensitivity/specificity assessment as well as cost/benefit analyses required for future implementation.

## Conclusion

To our knowledge, this is the first study to characterize both retinal structure and function in 5xFAD mice over a broad range of ages. We show that retinal neurodegeneration associated with amyloid pathology follows a progressive pattern similar to that of the brain. Amyloid pathology in the eye leads to neurodegeneration of ganglion cell structure (particularly their axons) and function, but interestingly a slight thickening of the synaptic layer between bipolar cells, amacrine cells, and ganglion cells. At the older ages, this was accompanied by more widespread retinal dysfunction encompassing the photoreceptors (a-wave), interneurons (b-wave) and the inner retina (oscillatory potentials). These data provide insight into specific patterns of early retinal changes and disease progression associated with amyloid pathology.

## Data Availability Statement

The original contributions presented in this study are included in the article/Supplementary Material, further inquiries can be directed to the corresponding author.

## Ethics Statement

The animal study was reviewed and approved by Howard Florey Institute Animal Experimentation Ethics Committee (Approval number 13-068-UM).

## Author Contributions

CN, BB, JL, and ZH conceptualized the study, designed the experiments, collected and analyzed the data, and wrote the manuscript. Q-XL and HC collected the data, analyzed the data, prepared the figures, and reviewed the manuscript. AV and JM conceptualized the study, designed the experiments, and reviewed the manuscript. All authors contributed to the article and approved the submitted version.

## Conflict of Interest

CN, BB, AV, and JM were joint investigators on an Australian Research Council Linkage grant LP160100126 with AstraZeneca Neuroscience and Biogen Inc. JM was an employee of AstraZeneca Neuroscience. The remaining authors declare that the research was conducted in the absence of any commercial or financial relationships that could be construed as a potential conflict of interest.
