# Supplementary material for: Retinal Functional and Structural Changes in the 5xFAD Mouse Model of Alzheimer’s Disease
Source: Front Neurosci. 2020 Aug 13;14:862. doi: 10.3389/fnins.2020.00862 (PMC7438734; doi:10.3389/fnins.2020.00862)
Supplement: Supplementary file 1 [file Data_Sheet_1.docx]

Supplementary Material

## Supplementary Figures

**Supplementary Figure 1.** **Component specific electroretinogram amplitudes as a function of luminous energy. Panels A – C** show P2 amplitudes. Inset shows amplitude at the dimmest light levels with a log y-axis to allow better appreciation of the pSTR amplitude. **Panels D – F** show OP amplitude as a function of luminous energy. *P2, bipolar cell response; pSTR, positive scotopic threshold response; OP, oscillatory potentials; all data shown, mean ± SEM.*
